# The Active Ingredients of Jiang-Zhi-Ning: Study of the *Nelumbo nucifera* Alkaloids and Their Main Bioactive Metabolites

**DOI:** 10.3390/molecules17089855

**Published:** 2012-08-16

**Authors:** Jianxin Chen, Xueling Ma, Kuo Gao, Yong Wang, Huihui Zhao, Hao Wu, Juan Wang, Hua Xie, Yulin OuYang, Liangtao Luo, Shuzhen Guo, Jing Han, Bing Liu, Wei Wang

**Affiliations:** Beijing University of Chinese Medicine, 11 Bei San Huan Dong Lu, ChaoYang District, Beijing 100029, China

**Keywords:** *Nelumbo nucifera*, alkaloids, Jiang-Zhi-Ning, substantial basis of Traditional Chinese Medicine, 2-hydroxy-1-methoxyaporphin

## Abstract

The object of this study was to identify the major active ingredients of the Chinese Traditional Medicine Jiang-Zhi-Ning (JZN) based on the high performance liquid chromatography (HPLC) profiles of plasma samples obtained from beagle dogs at different times after intragastric administration of JZN, crude JZN extracts, different extracted fractions, different subfractions of the active fraction and different isolated ingredients. 2-Hydroxy-1-methoxyaporphin (**2H1M**), an alkaloid from *Nelumbo nucifera*, one of the herbs that make up JZN, was identified as the constituent showing the major pharmacodynamic effect. The major metabolites of **2H1M** were analyzed and identified as *N*-demethyl-2-hydroxy-1-methoxyaporphine-2-*O*-glycuronic acid, 2-hydroxy-1-methoxy-aporphine-2-*O*-glycuronic acid and 2-hydroxy-1-methoxy-aporphine-2-*O*-sulphate. This study provided a comprehensive insight into the active components of JZN.

## Abbreviations

DSC: differential scanning calorimetry
HL: hyperlipidemia
HPLC: high performance liquid chromatography
JZN: Jiang-Zhi-Ning
LC/MS: liquid chromatography–mass spectrometry
2H1M: 2-hydroxy-1-methoxyaporphin
UV: ultra-violet spectrophotometry

## 1. Introduction

The Traditional Chinese Medicine Jiang-Zhi-Ning (JZN), contains four kinds of Chinese medicinal herbs: *Polygonum multiflorum*, *Fructus crataegi*, *Nelumbo nucifera* and *Semen cassiae*. Abundant Chinese literature has shown that JZN is able to enhance blood circulation of the coronary arteries, reduce arrhythmia and significantly reduce the levels of blood lipids [1,2,3,4,5]. Research has been focused on the mechanism of action of JZN, while the identity of the active materials responsible for JZN’s activity remains unclear. Our team has focused on the pharmacodynamics of JZN, confirming that JZN had reliable lipid-lowering and antioxidative effects [1,2].

*Nelumbo nucifera* is one of the main herbs in JZN and alkaloids are its main active constituents. Studies of the alkaloids of *Nelumbo nucifera* date back to the 19th century when nuciferine, *O*-nornuciferine and roemerine were isolated from the plant. Based on the structure of their nuclei, the *Nelumbo nucifera* alkaloids can be divided into three categories: single benzylisoquinolines, aporphines and dehydrogenated aporphines. The aporphine class includes nuciferine [6], *N*-nornuciferine [7], *O*-nornuciferine [7], liriodenine [8], roemerine [9], *N*-norarmepavine [10] and 2-hydroxy-1-methoxyaporphine [9]. Many researchers have showed that the alkaloids from *Nelumbo nucifera* have lipid-lowering, weight-loss inducing, antibacterial, and antiviral functions [11,12].

## 2. Results and Discussion

### 2.1. Identification and Attribution of the Effective Components of JZN

**Figure 1:**
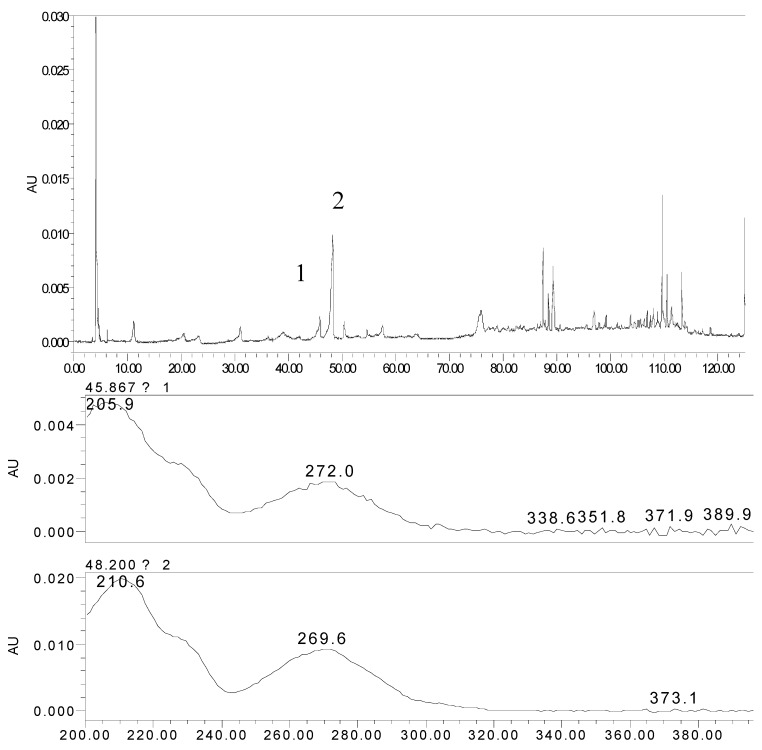
HPLC and UV spectra of the plasma sample after intragastric administration of extract of JZN.

**Figure 2:**
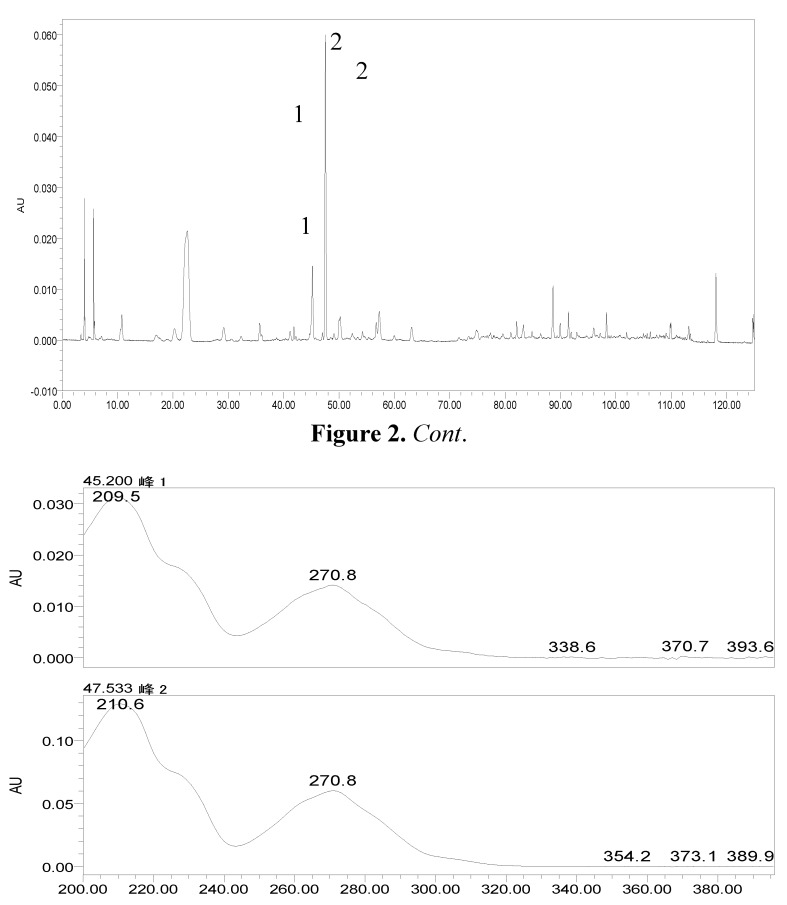
HPLC and UV spectra of the plasma sample after intragastric administration of the extract of *Nelumbo nucifera*.

**Figure 3:**
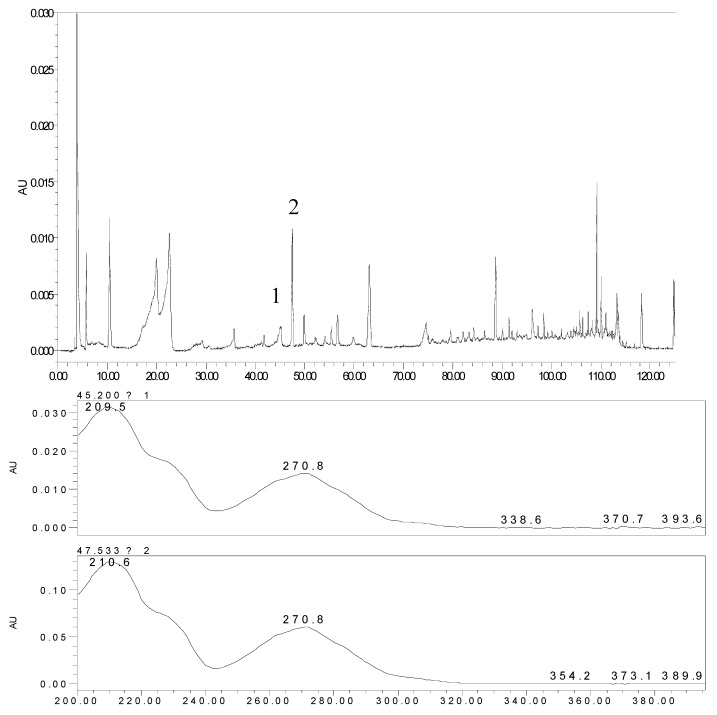
HPLC and UV spectra of the plasma sample after intragastric administration of the alkaloids of *Nelumbo nucifera*.

**Figure 4:**
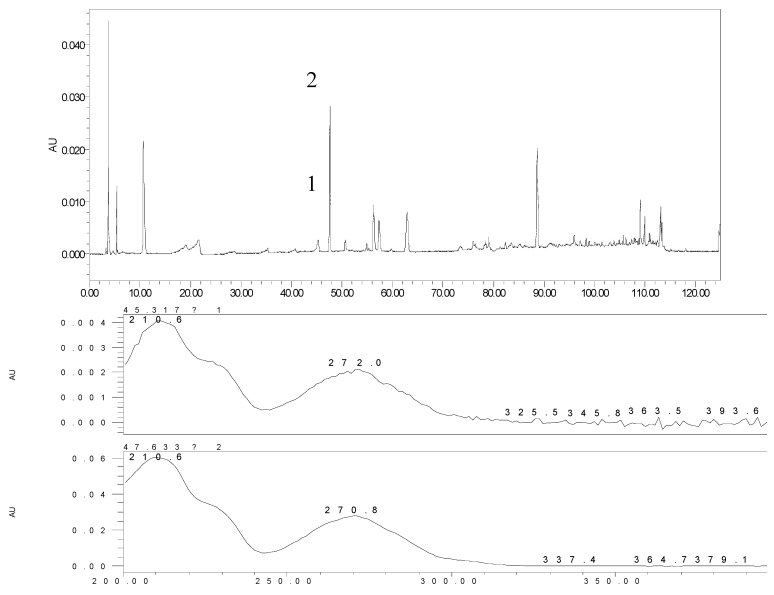
HPLC and UV spectra of the plasma sample after the intragastric administration of 2-hydroxy-1-methoxyaporphine.

**Figure 5:**
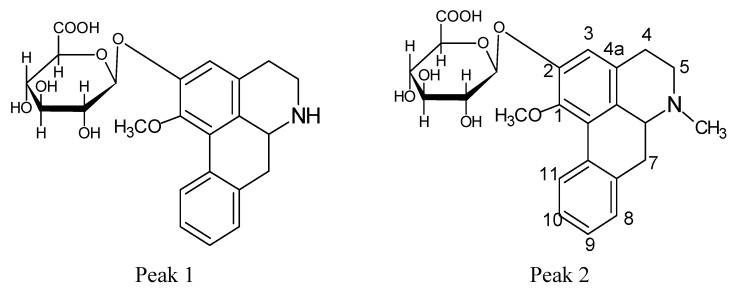
Structures of effective metabolites in JZN.

**Figure 6:**
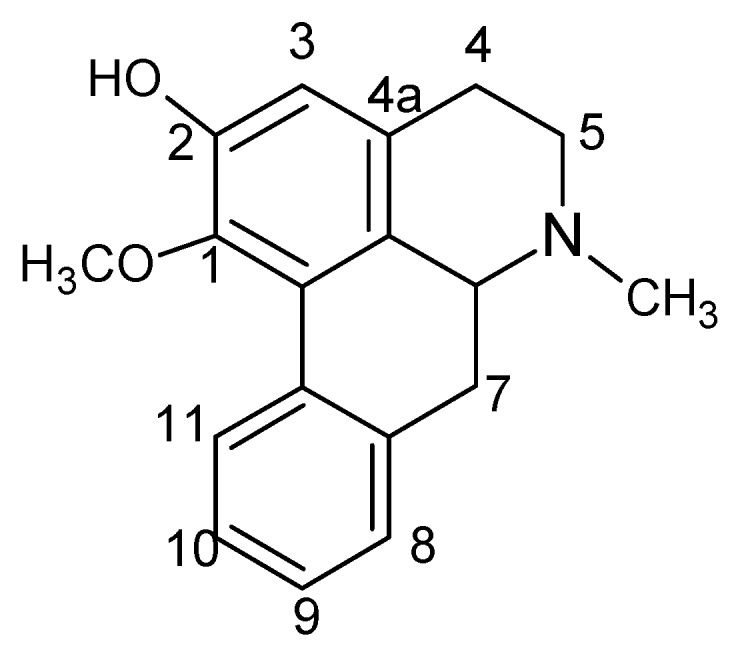
Structure of **2H1M**.

In order to provide a comprehensive insight into the active materials of JZN, serum chemical methods have been used, starting with the compounds absorbed into blood, to clarify the pharmacodynamic substances of JZN present in the body, the original precursors of the major active ingredients and their relation to the alkaloid composition of *Nelumbo nucifera*. Extract of JZN, crude drug extracts, different extracted fractions, and different ingredients were administered intragastrically to beagle dogs. Blood samples were taken 1 h later, the plasma was processed as described in the Experimental and the various samples were then examined by HPLC. Two peaks, identified in the chromatograms as peaks 1 and 2 appeared in the traces of samples taken after administration of extract of JZN (Figure 1), extract of *Nelumbo nucifera* (Figure 2), the alkaloids of *Nelumbo nucifera* (Figure 3) and 2-hydroxy-1-methoxyaporphine (Figure 4). Through fingerprint comparisons and subsequent structure elucidation by a variety of techniques described below we identified peaks 1 and 2 as the two metabolites shown in Figure 5, which in turn led to the identification of their precursor as 2-hydroxy-1-methoxyaporphine (Figure 6), an known alkaloid found in *Nelumbo nucifera*.

### 2.2. The Metabolites of ***2H1M***

**Figure 7:**
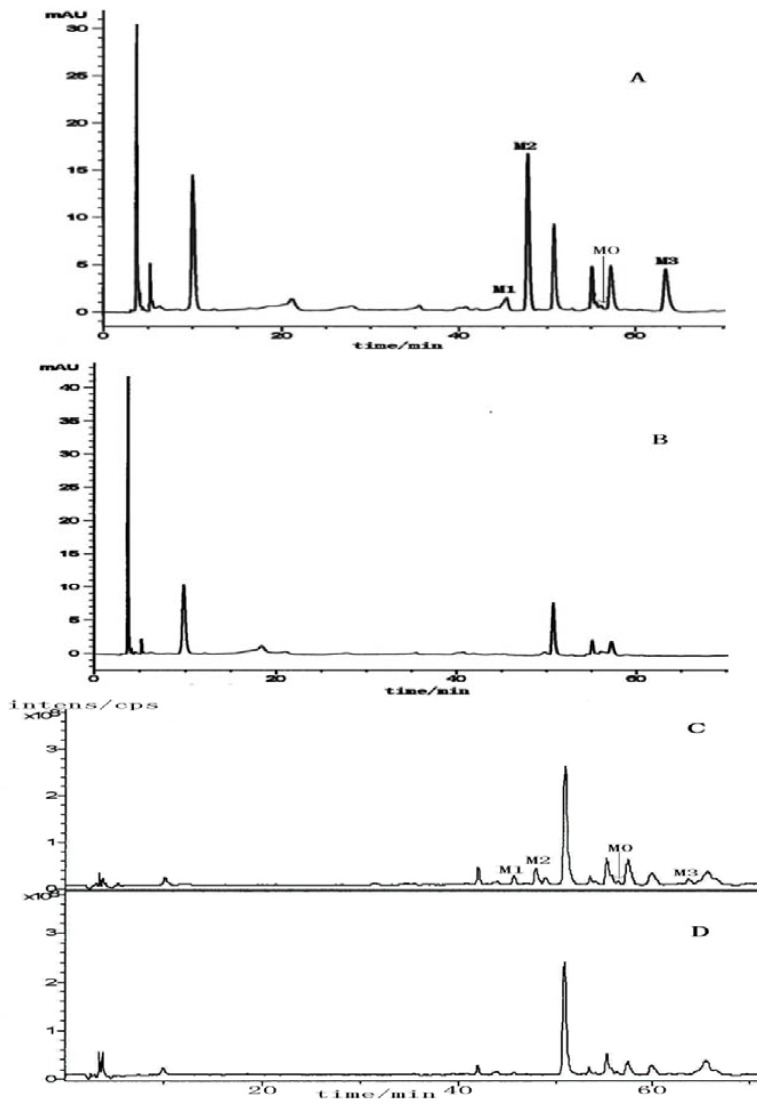
HPLC traces and total ion chromatograms of **2H1M** and its metabolites in the plasma of beagle dogs. (**A**) HPLC of the plasma sample; (**B**) HPLC of the blank plasma; (**C**) total ion chromatograms of the plasma sample; (**D**) total ion chromatograms of the blank plasma; M0: **2H1M**; M1~M3: metabolites of **2H1M**.

By LC/MSn analysis we obtained the HPLC traces and total ion chromatograms of the blank sample and plasma samples after intragastric administration of the drug for 1 h (Figure 7). Peak M0 corresponded to **2H1M** and peaks M1, M2, M3 were from metabolites.

**Figure 8:**
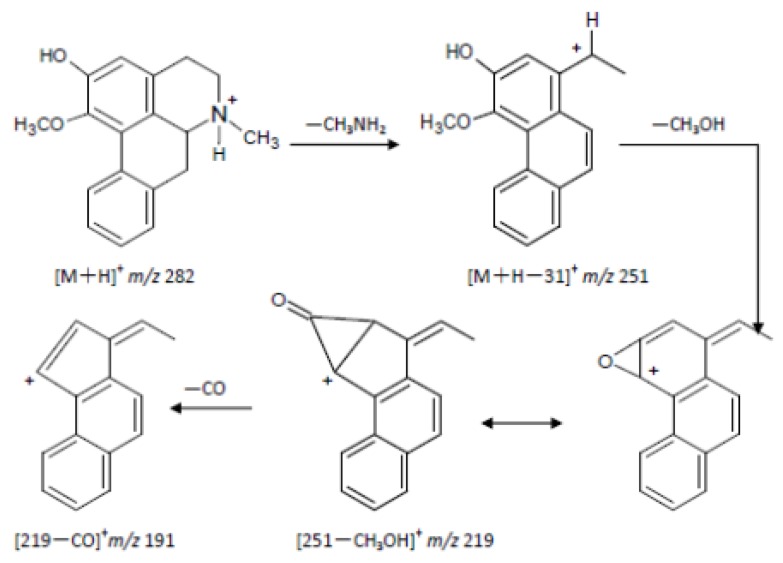
Fragmentation pathway of **2H1M**.

**Figure 9:**
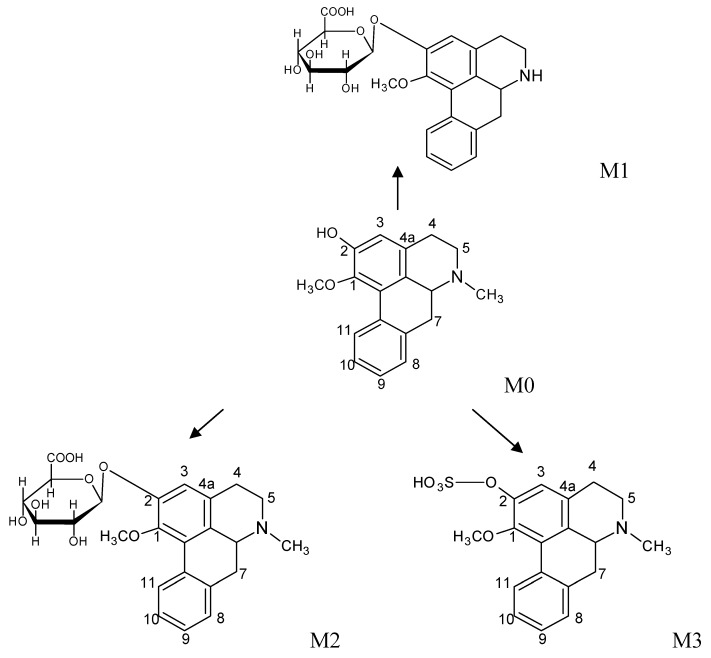
The main metabolic pathway of **2H1M** (M0); M1~M3–phase II metabolites.

Through comprehensive analyses of retention times, first and second ESI fragment information, comparisons with reference substances and structure analyses, we reached the following conclusions: the quasi-molecular ion [M+H]^+^ of M0 (retention time 56.3 min) in the primary full-scan mass spectrum was located at *m/z* 282. Fragment ions of *m/z* 251 ([M+H−CH3NH2]^+^), 219 ([251−CH3OH]^+^) and 191 ([219−CO]^+^) were obtained in the secondary MS. M0 and a **2H1M** reference sample had the same chromatographic MS behavior, so the authors concluded that the M0 was **2H1M**. Its fragmentation pathways are shown in Figure 8 and Figure 9.

**Figure 10:**
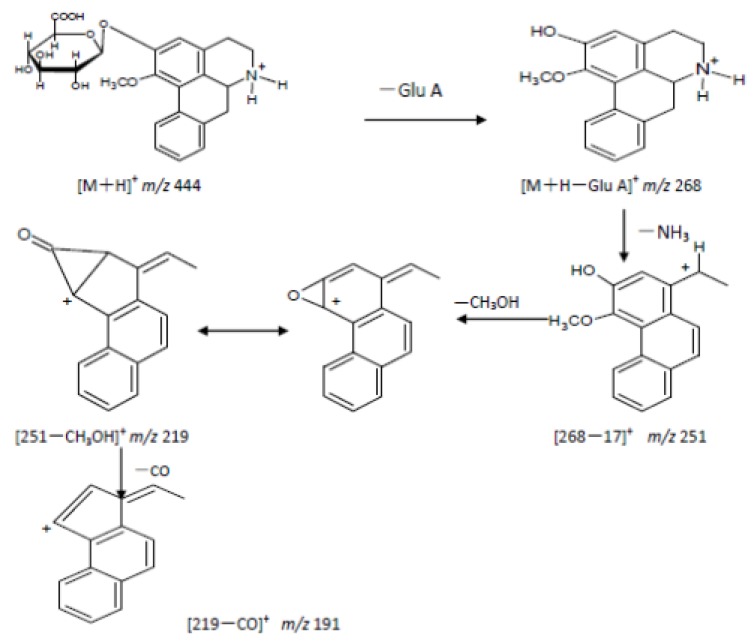
Fragmentation pathway of M1.

M1: the retention time was 45.6 mins, the quasi-molecular [M+H]^+^ in the primary full-scan mass spectrum was *m/z* 444, and a fragment of *m/z* 268 [M+H−176]^+^ was obtained in the secondary MS, which corresponds to the loss of a glucuronic acid moiety. The fragment ion at *m/z* 268 is 14 amu less than 2-hydroxy-1-methoxyaporphine’s quasi-molecular ion. Supposing that **2H1M** was demethylated, the metabolite with a glucuronic acid appeared at the same time. Fragment ions at *m/z* 268, 251, 219, 191 were obtained in the secondary MS. Comparing with 2-hydroxy-1-methoxyaporphine’s quasi-molecular ion at *m/z* 282, we noted that a fragment ion for [268−NH3]^+^ appeared, but the fragment ion of [268−CH3NH2]^+^ had disappeared. Based on the fragmentation regularity, if there was methyl attached at the N position, there would be a [M+H−CH3NH2]^+^ fragment, and when the methyl was replaced by a hydrogen ion, there would be a [M+H−NH_3_]^+^ fragment [13], so the authors concluded that M1 was the adduct of *N*-demethylated 2-hydroxy-1-methoxyaporphine and 2-*O*-glucuronic acid. Its fragmentation pathways are shown in Figure 10.

M2: the retention time was 47.9 min and the quasi-molecular [M+H]^+^ in the primary full-scan mass spectrum was *m/z* 458. The fragment ion at *m/z* 268 is 176 less than M0’s quasi-molecular ion, indicated that a glucuronic acid moiety was added to the precursor in the metabolite. Fragmentation ions at *m/z* 282, 251, 219, 191 were obtained in the secondary MS and the fragment ion of *m/z* 282 corresponded to the fragment of the quasi-molecular ion at *m/z* 458 without a glucuronic acid. Therefore, M2 was considered as the combination of **2H1M** and a glucuronic acid moiety. The structure is shown in Figure 9.

M3: the retention time was 63.6 min, the quasi-molecular ion [M+H]^+^ in the primary full-scan mass spectrum was seen at *m/z* 362. The fragment ion at *m/z* 268 was 176 less than M0’s quasi-molecular ion (the sulfate fragment ion was *m/z* 80), suggesting that metabolism had formed the sulfate of the precursor molecule. Fragment ions at *m/z* 282, 251, 219, 191 were obtained in the secondary MS. The fragment ions were all precursor fragments, therefore M3 was identified as the adduct of **2H1M** and a sulfate. The structure is shown in Figure 9.

All three kinds of metabolites were phase II metabolites: M1 was the result of **2H1M** *N*-demethylation/2-*O*-glucuronic acid conjugation. M2 was the conjugate of **2H1M** and glucuronic acid. M3 was the conjugate of **2H1M** and sulfate. 2-Hydroxy-1-methoxyaporphin (**2H1M**), an alkaloid from *Nelumbo nucifera* expressing major pharmacodynamic action, was confirmed as the endosomatic constituent. 

Automated LC/MS technology has become a routine analysis method, playing an ever more important role in Chinese medicine and studies of natural metabolite kinetics. LC/MS is able to not only avoid clumsy, time-consuming and complicated process of separation and purification, but also detect and identify the traces of drugs rapidly and conveniently [14]. General metabolic pathways of Chinese medicines *in vivo* are the processes of oxidation, reduction and hydrolysis, forming the high polarity phase I metabolites, or conjugation with uronic acid, amino acids, *etc.*, forming the phase II metabolites. We used LC/MS to analyze the *in vivo* metabolism of **2H1M** for the first time, and found three kinds of phase II metabolites. The results provided a reasonable basis for further research and laid a good foundation for the research on the regularity of 2-hydroxy-1-methoxyaporphine’s metabolism *in vivo*. At present, the *in vivo* metabolism of nitrogen compounds has drawn great attention. The reason is that metabolites with great reactivity and toxicity could be obtained by this pathway [15]. The possibility of one of the metabolites of **2H1M** (combination of **2H1M** and *N*-demethylation/2-*O*-glucuronic acid) undergoing nitrosation and producing toxicity must be investigated as a next step.

## 3. Experimental

### 3.1. General

Instruments used: Waters 1525 high performance liquid chromatograph, Waters 2996 PAD detector, Agilent 1100 Series LC/MSn instrument, including quaternary gradient pump, automatic injector, column oven, DAD detector and MSD Trap XCT ion trap mass spectrometer (equipped with electrospray ionization source); Boetius PHMK 05 micro melting point detector; VG-ZAB-HS mass spectrometer; Bruker Avance DRX-500 superconducting NMR; Shimadzu DSC-60 differential scanning calorimeter; TU-1810 APC UV-Vis spectrophotometer; ZF-I ultraviolet analyzer; KQ-500DE ultrasonic cleaner; Sartorious BT 25S Type 1/100000 electronic analytical balance; QL-901 vortex mixer; Cleaner C18 the SPE solid phase extraction column 

### 3.2. Identification and Attribution of Effective Ingredients of JZN

#### 3.2.1. Experimental Animals and Sample Preparation

Healthy adult beagle dogs weighing 10 ± 1 kg were purchased from the Beijing Tongli Experimental Animal Facility and raised in the standard animal facility at the university. All experimental protocols were approved by the ethics committee of Beijing University of Chinese Medicine. Plasma (2 mL) was extracted using Cleaner C_18_ Solid Phase Extraction technology (3 mL, 200 mg, 60 µm, activated by 5 mL methanol and then balanced by 5 mL water), and each solid phase extraction cartridge was eluted with water (5 mL) and methanol (2 mL). The methanol eluate was collected and evaporated to dryness with cold air in a water bath at 50 °C. It was then concentrated to 0.5 mL and filtered through a 0.45 µm microfiltration membrane. The blank control sample was prepared in the same way. One hundred µL samples were used for HPLC analysis.

#### 3.2.2. Chromatographic Conditions

Chromatograms: Agilent TC-C18 (4.6 mm × 250 mm, 5 µm), Extend-C_18_ guard column; column temperature: 30 °C; flow rate: 1.0 mL/min; detection wavelength: 200–400 nm; mobile phase: acetonitrile (A)-0.01% formic acid (B). Gradient elution conditions are listed in Table 1.

**Table 1:** Gradient elution conditions.

Time (min)	Acetonitrile (%)	Water (0.01% Formic acid) (%)
0	2	98
20	2	98
50	16	84
65	16	84
90	40	60
100	60	40
110	100	0

#### 3.2.3. Source of Precursor and Identification

Preparations of extracts of JZN: (1) *Polygonum multiflorum* (25 g) and *Nelumbo nucifera* (75 g) were mixed and put in a 25-fold excess of 50% ethanol. After heating under reflux for 1.5 h, obtained the ethanol extract and the residues were ﬁltered and distilled again for another two times in the same way. The three ethanol extracts were combined and concentrated to a paste, which is the whole solids I. (2) *Fructus crataegi* (500 g) and *Semen cassiae* (25 g) were mixed and put in a 7-fold excess of water. After heating at reflux for 2.0 h, obtained the aqueous extract and the residues were ﬁltered and distilled another time in the same way. The two aqueous extracts were combined and concentrated to a paste, which are the whole solids II. The two whole solids were mixed to obtain the extract of JZN with a yield of 37% after drying under vacuum at 50 °C.

Preparations of extracts of *Nelumbo nucifera* and preparation of *Nelumbo nucifera* alkaloid extracts: *Nelumbo nucifera* in the proportion of the compound recipe was weighed out to give the extract of *Nelumbo nucifera*. Next a portion of *Nelumbo nucifera* was taken up in a 16 fold-excess of 90% ethanol. After reflux extraction for 1.5 h, obtained the ethanol extract and the residues were ﬁltered and distilled another two times in the same way. The three ethanol extracts were combined and solvent was removed under reduced pressure to dryness. The residue was dissolved in 1% HCl (10 fold the amount *Nelumbo nucifera*), dispersed by ultrasound (30 min, 100 Hz) and centrifuged (30 min, 3,000 r/min). The supernatant was set aside and the procedure repeated again with the residue. The combined supernatants was added onto a D00-cc macroporous cation exchange resin column (the ratio of diameter to height was 1:8), and adsorption flow rate was 10 BV/h until the saturation adsorption. Impurities were eluted with 50% ethanol (5 BV) and 1% ammonia and ethanol solution. The solvent was recovered by decreasing the pressure and the residue was taken to dryness by decreasing the pressure to obtain the alkaloid extract. The extract of JZN, extract of *Nelumbo nucifera* and alkaloids of *Nelumbo nucifera* were irrigated into beagle dogs. Blood samples were taken 1 h later.

#### 3.2.4. Attribution of Precursors in Different Alkaloid Fractions of *Nelumbo nucifera*

The alkaloid part of *Nelumbo nucifera* (50 g) was fractionated by silica gel column chromatography (160–200, 200 g), eluting with chloroform, chloroform-methanol (1:1) and methanol, to give 24 g of chloroform eluate, 16.87 g of chloroform-methanol (1:1) eluate and 0.34 g of methanol eluate. Alkaloids were only found in the chloroform and chloroform-methanol (1:1) fractions. According to the proportion of the *Nelumbo nucifera* in the compound recipe, the authors took 0.056 g of eluate from the chloroform fraction and 0.0374 g of eluate from the chloroform-methanol (1:1) fraction, added distilled water, and used ultrasound to get a suspension which was administered to the beagle dogs and blood examples were collected at the one hour point.

Chloroform eluate (0.0026 g), chloroform-methanol (1:1) elute (0.0038 g) and methanol eluate (0.0030 g) were separately put into a 5 mL volumetric flask, dissolved in methanol, diluted to the scale and then filtered through a 0.45 μm microfiltration membrane. The HPLC of each eluate was obtained according to the mentioned analysis frame under the chromatography items. After comparing the HPLC chromatograms obtained from the different eluates, we could be assured that the precursor of peak1 and peak 2 came from the chloroform eluate.

#### 3.2.5. Structure Identification of **2H1M**

The analytical data of **2H1M** was as follows: melting point 195–197 °C, UV λ_max_ (nm): 272. EI-MS: *m/z* 281 [M]^+^, 266 [M−CH_3_]^+^, 251 [M−2CH_3_]^+^, 250, 178. ^1^H-NMR (CDCl_3_, 500 MHz): δ 8.27 (H, d, *J* = 8.0 Hz, OH), 7.25, 7.26, 6.60 (hydrogen in the benzene ring), 3.56 (3H, s, O-CH_3_), 3.11 (2H, t, H-5), 3.01 (2H, t, H-7), 2.68 (2H, t, H-4), 2.55 (3H, s, N-CH_3_), 2.48 (1 H, d, J = 4.0, 8.0 Hz, H-6a). ^13^C-NMR (CD_3_OD, 125 MHz): δ 147.9 (C-2), 143.0 (C-1), 136.3 (C-7a), 131.8 (C-11a), 128.0 (C-8), 127.3 (C-1a), 127.2 (C-9), 127.2 (C-1b), 127.3 (C-11), 127.3 (C-4a), 125.8 (C-10), 114.1 (C-3), 62.4 (C-6a), 60.3 (OCH_3_), 53.3 (C-5), 43.8 (N-CH_3_), 34.8(C-7), 28.7 (C-4).

### 3.3. Experiments Investigating the Metabolites of ***2H1M***

#### 3.3.1. Isolation of **2H1M**

*Nelumbo nucifera* 5kg were mixed with a 16-fold excess of 90% ethanol. Samples were heated and refluxed three times and each for 1 h. After removal of the solvent to dryness the residue was dissolved in 1% HCL (25-fold the amount of *Nelumbo nucifera* extract residue), dispersed by ultrasound (30 min, 100 Hz) and centrifuged (30 min, 3000 r/min). The supernatant was adjusted with 1 mol/L NaOH to pH = 7.5 and extracted with chloroform. The extract was cocentrated to recover the chloroform until the solution was dry. The residue was separated by silica gel column chromatography, and eluted with ligroin-acetone (2:1). The eluate was purified by alumina column chromatography, eluted with ligroin- acetone (4:1) to give white and acerous crystals of **2H1M**.

#### 3.3.2. Plasma Sample Collection and Preparation

Three healthy beagle dogs, weight (10 ± 1) kg, were not fed any food for 18 h before the experiments. **2H1M** was given to the beagle dogs as a level of 1.5 mg·kg^−1^. After 60 min a 5 mL blood sample was obtained from the brachial vein. The blood sample, treated with the anti-coagulant heparin, was centrifuged for 30 min (3,000 r/min) and the plasma obtained was kept at a temperature of −40 °C until analyzed.

Plasma (1 mL) was extracted by Cleanert C18 Solid Phase Extraction technology (3 mL, 200 mg, 60 µm, activated by 5 mL methanol and then balanced by 5 mL water), and each solid phase extraction cartridge was eluted by 5 mL water and 2 mL methanol. The methanol eluent was collected and evaporated to dryness with cold air at room temperature. The residue was dissolved in methanol (500 µL) to give the plasma sample.

#### 3.3.3. LC/MSn Conditions

Chromatographic process: sample size: 100 µL; column: Agilent TC-C_18_ (4.6 mm × 250 mm, 5 μm); column temperature: 30 °C; rate: 1.0 mL·min^−1^; detection wavelength: 272 nm; mobile phase: acetonitrile (A) — 0.01% aqueous formic acid (B). Gradient elution: eluent A was 2% in 0~20 min and increased from 2% to 16% in 20~50 min.

MS: ESI Conditions: inspection method: positive ion; atomization pressure: 40 KPa; flow rate of dry gas: 10 L·min^−1^; temperature of dry gas: 350 °C; spray voltage: 4 kV; multi-level scanning collision gas: nitrogen.

## 4. Conclusions

In this research, we studied JZN’s pharmacodynamic constituents as well as the structure of its metabolites. The results showed that **2H1M**, an alkaloid from *Nelumbo nucifera*, was an endosomatic constituent, responsible for the major pharmacodynamic action. The metabolites of **2H1M** were identified as 2-hydroxy-1-methoxyaporphine-*N*-demethyl-2-*O*-glycuronic acid, 2-hydroxy-1-methoxy-aporphine-2-*O*-glycuronic acid and 2-hydroxy-1-methoxyaporphine-2-*O*-sulphate. This study provided a comprehensive insight into the substantial materials of JZN.
